# Structure-Based Design of Biologically Active Compounds

**DOI:** 10.3390/molecules25143115

**Published:** 2020-07-08

**Authors:** Sandra Gemma

**Affiliations:** Department of Biotechnology, Chemistry and Pharmacy, University of Siena, via Aldo Moro 2, 53100 Siena, Italy; gemma@unisi.it

The past decades have witnessed tremendous progress in the detailed structural knowledge of proteins as potential or validated drug targets and the discovery of new drugs based on this wealth of knowledge progressed in parallel.

In this Special Issue, several papers deal with the structure-based investigation of targets useful for the discovery of novel anticancer drugs. Al-Warhi et al. [1] targeted the serine/threonine protein kinase CDK4, and using a molecular hybridization strategy designed a series of oxindole–indole conjugates that displayed interesting anti-proliferative activity. Ziemska et al. [2] performed an in silico study where they selected from commercial databases the 3,4-dihydroisoquinoline scaffold for the identification of potential inhibitors of the enzyme leucine aminopeptidase. Khan et al. [3] designed and synthesized copper (II) 2-hydroxy-1-naphthaldehyde complexes and investigated their anticancer activity in depth. In this study, emerged the capability of the copper complexes under investigation to behave as multitargeting agents able to interfere with different pathways, including reactive oxygen species (ROS) production, induction of apoptosis and autophagy.

Targets involved in both cancer and inflammatory diseases have been the focus of intense structure-based design campaigns and are well-represented in this Special Issue. Londhe et al. [4] analyzed four X-ray structures of keap1-Nrf2 inhibitors using molecular dynamics (MD) simulations and the path-based free energy method of umbrella sampling (US) for deriving amino acid residues involved in hydrophobic and electrostatic interactions useful for structure-based design of improved inhibitors. Lee et al. [5] analyzed the binding pocket of autotaxin by molecular dynamics and investigated the role of the water molecules in the binding site through a topological water network (TWN) analysis. Based on the results of this analysis, they successfully designed and synthesized novel and potent autotaxin ligands. Yan et al. [6] targeted the cyclooxygenase-2 (COX-2) enzyme through the synthesis of a novel series of potent and selective COX-2 inhibitors that displayed antiproliferative activity in vitro. The most potent compound also showed anti-colon cancer activity in a SW620 xenograft mouse model.

Two papers of this Special Issue deal with covalent ligands and the challenges posed by in silico analysis of covalent inhibitors. Wagner et al. [7] designed and synthesized a covalent histamine H_3_ receptor (H_3_R) ligand, bearing an isothiocyanate warhead, that behaved as an inverse agonist of this G protein-coupled receptor (GPCR). Scarpino et al. [8] designed nonpeptidic covalent inhibitors of the β5i subunit of the immunoproteasome bearing a boronic acid moiety. Both noncovalent and covalent docking procedures were included in their screening workflow performed on an ad-hoc compiled library of boronic acid derivatives.

Based on the concept that the knowledge of interactions at either a molecular or high topological level are important for the design of effective ligands, Bojarska et al. [9] designed and synthesized a novel ornithine derivative and analyzed the molecular and supramolecular structure.

The design of anti-infective compounds and of pest control agents is another field in which structure-based design plays a critical role for both the discovery of new agents and for the control of drug-resistance issues. Tian et al. [10] analyzed the mutations induced on the ionotropic gamma-aminobutyric acid (GABA) receptor, in insects presenting resistance to the insecticide fipronil, and their studies could be used for the design of improved insecticides. In a structure-based virtual screening campaign of FDA approved drugs performed on two targets (zmp1 and peptide deformylase -PDF-) relevant for *Mycobacterium tuberculosis* survival and virulence, Battah et al. [11] identified a number of potential multitargeting drugs displaying interesting activity in vitro as antitubercular agents and suitable for repurposing approaches in the therapy of this deadly infection. Finally, Battista et al. [12] provided a comprehensive overview of the structural information available on trypanothione reductase, a highly promising drug target for the development of innovative therapies against trypanosomiases and leishmaniasis.
